# Epigenetic and transcriptional regulation of cytokine production by *Plasmodium falciparum*-exposed monocytes

**DOI:** 10.1038/s41598-024-53519-w

**Published:** 2024-02-05

**Authors:** David V. L. Romero, Thivya Balendran, Wina Hasang, Stephen J. Rogerson, Elizabeth H. Aitken, Adrian A. Achuthan

**Affiliations:** 1 Department of Medicine, Royal Melbourne Hospital, The University of Melbourne, 1F Royal Parade, Parkville, VIC 3010 Australia; 2 Department of Infectious Diseases, The University of Melbourne, The Peter Doherty Institute for Infection and Immunity, Melbourne, VIC Australia; 3 Department of Microbiology and Immunology, The University of Melbourne, The Peter Doherty Institute for Infection and Immunity, Melbourne, VIC Australia

**Keywords:** Inflammation, Malaria

## Abstract

*Plasmodium falciparum* infection causes the most severe form of malaria, where excessive production of proinflammatory cytokines can drive the pathogenesis of the disease. Monocytes play key roles in host defense against malaria through cytokine production and phagocytosis; however, they are also implicated in pathogenesis through excessive proinflammatory cytokine production. Understanding the underlying molecular mechanisms that contribute to inflammatory cytokine production in *P. falciparum*-exposed monocytes is key towards developing better treatments. Here, we provide molecular evidence that histone 3 lysine 4 (H3K4) methylation is key for inflammatory cytokine production in *P. falciparum*-exposed monocytes. In an established in vitro system that mimics blood stage infection, elevated proinflammatory TNF and IL-6 cytokine production is correlated with increased mono- and tri-methylated H3K4 levels. Significantly, we demonstrate through utilizing a pharmacological inhibitor of H3K4 methylation that TNF and IL-6 expression can be suppressed in *P. falciparum*-exposed monocytes. This elucidated epigenetic regulatory mechanism, controlling inflammatory cytokine production, potentially provides new therapeutic options for future malaria treatment.

## Introduction

Malaria is responsible for around 625,000 deaths and 245 million cases annually, with pregnant women and children under five in sub-Saharan Africa being most highly impacted^1^. The majority of the deaths is caused by infection with *Plasmodium falciparum*, and these deaths are characterized by excessive cytokine production by immune cells that sense and phagocytose *P. falciparum*-infected red blood cells (iRBC) and parasitic components, such as hemozoin (Hz)^2^. Primary immune cells that respond to iRBC and trigger subsequent immune responses include monocytes^3,4^. Detection and subsequent phagocytosis of malarial antigens by monocytes trigger downstream signaling pathways leading to activation of transcription factors, such as NF-kB, that subsequently regulate cytokine production^5^. Activated monocytes are known to produce pro-inflammatory cytokines, including tumor necrosis factor (TNF) and interleukin-6 (IL-6), that facilitate the clearance of parasites by recruiting immune cells to sites of infection^6^, increasing cell-to-cell interaction^7^, and promoting the differentiation of naïve lymphocytes into their adaptive immune cell counterparts^8^ to control parasitemia.

While pro-inflammatory cytokines are critical to control parasitemia, their excessive production can cause disease. By upregulating the expression of same adhesion molecules that infected cells bind to endothelial cells, excess TNF and IL-6 also promote the cytoadherence of iRBC^9^. This results in more transmigration of leukocytes and additional inflammatory cytokine secretion, thus creating a harmful cycle^10^. The detrimental effects of this cytoadherence cycle are exacerbated once iRBC bind in vital organs such as brain or lungs, leading to cerebral malaria or acute lung injury^11–14^. To counteract the pro-inflammatory functions of TNF and IL-6, monocytes produce anti-inflammatory cytokines, such as IL-10, which can suppress pro-inflammatory cytokine production by limiting NF-kB expression and desensitizing the cell responses^15^.

A new layer of parasitic immune regulation has been emerging in the form of epigenetic histone modifications, which can dictate the accessibility of gene promoter regions to transcription factors, thereby controlling gene expression^16^. It is known that NF-kB induces JMJD3, a JmjC family histone demethylase, to remove the repressive histone 3 lysine 27 trimethylation mark (H3K27me3) to promote gene transcription of TNF and IL-6 in lipopolysaccharide (LPS)-treated monocytes^17,18^. Although it is widely recognized that overproduction of pro-inflammatory cytokines is a key process contributing to the pathogenesis of malaria, the underlying molecular mechanisms in *P. falciparum-*exposed monocytes are not well understood. In addition, the epigenome of *P. falciparum* has been extensively studied to exploit antimalarial properties^19^ but the histone modifications that may contribute to cytokine production in monocytes exposed to iRBC have not been well explored.

In the present study, we established an in vitro monocyte system to directly investigate the impact of *P. falciparum* on monocytes at the transcriptional and epigenetic levels. We found that lysed *P. falciparum*-iRBC promoted production of TNF and IL-6 in monocytes. However, the increased production of the pro-inflammatory cytokines resulted in only a marginal increase in NF-kB activity but no decrease in repressive H3K27me3. A broad screening of H3 modifications identified mono- and tri-methylated H3K4 in *P. falciparum*-exposed monocytes. Significantly, through pharmacological inhibition of H3K4 methylation, we demonstrated that TNF and IL-6 formation could be attenuated in *P. falciparum*-exposed monocytes. Given the association of excessive pro-inflammatory cytokine production with malaria pathology, elucidating regulatory mechanisms that control their production in *P. falciparum*-exposed monocytes may provide new targets for future malaria therapies.

## Results

### Lysed *P. falciparum*-infected RBC promote significant levels of TNF and IL-6 cytokine production by monocytes

To investigate the impact of *P. falciparum* blood-stage parasites on circulating monocytes at the transcriptional and epigenetic levels, an in vitro system was established. Isolated monocytes from healthy human donors were treated with live *P. falciparum* infected red blood cells (Live iRBC), lysed infected red blood cells (Lysed iRBC), or hemozoin (Hz) at a ratio of 10 iRBC or 10 iRBC-equivalent of Hz per monocyte for 4 and 24 h. TNF, IL-6 and IL-10 cytokine mRNA and protein expression were measured by qPCR and ELISA, respectively.

**Figure 1:**
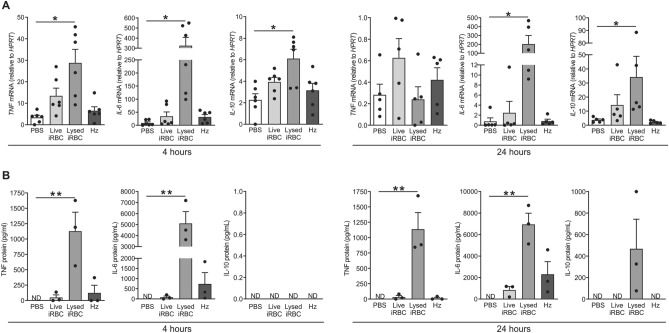
Lysed *P. falciparum*-infected RBC promote significant levels of TNF and IL-6 cytokine production in monocytes. Human monocytes (1 × 10^5^) were exposed to either live *P. falciparum*-infected red blood cells (10 live iRBC per monocyte), lysed infected red blood cells (10 lysed iRBC per monocyte), hemozoin (10 iRBC worth of Hz per monocyte) or left untreated (PBS) for 4 and 24 h. (**A**) *TNF, IL-6 and IL-10* mRNA expression measured by qPCR with *HPRT* as internal control (n = 5 donors). (**B**) Secreted TNF, IL-6 and IL-10 cytokines measured by ELISA (n = 3 donors). Results are shown as scatter plots with error bars indicating mean ± SEM. P-values were obtained using paired one-way ANOVA, using Dunnett’s multiple comparison test to compare each condition to the untreated control, where *P < 0.05, **P < 0.01. *ND* not detected.

Among these three different malaria stimuli, the lysed iRBC-exposed monocytes produced significant levels of all three cytokine transcripts at 4 h (Fig. 1A). At 24 h, the mRNA expression of TNF was similar to baseline, but IL-6 and IL-10 levels remained elevated. Consistent with the mRNA expression at 4 h, significant levels of secreted TNF and IL-6 were measured in the culture supernatants of monocytes exposed to iRBC, but IL-10 could not be detected (Fig. 1B). Secreted TNF and IL-6 proteins remained elevated 24 h post-exposure to iRBC. While secreted IL-10 could be measured in iRBC-exposed monocyte culture supernatants at 24 h, the levels were not significantly higher than PBS control. Given our finding that only lysed iRBC was able to activate a robust cytokine production, both at mRNA and protein levels, as early as 4 h, we utilized this optimized in vitro system in all subsequent experiments to investigate the transcriptional and epigenetic regulation of cytokine production by monocytes exposed to *P. falciparum*.

### Lysed *P. falciparum*-infected RBC marginally regulate NF-kB activity but not H3K27 modification in monocytes

**Figure 2:**
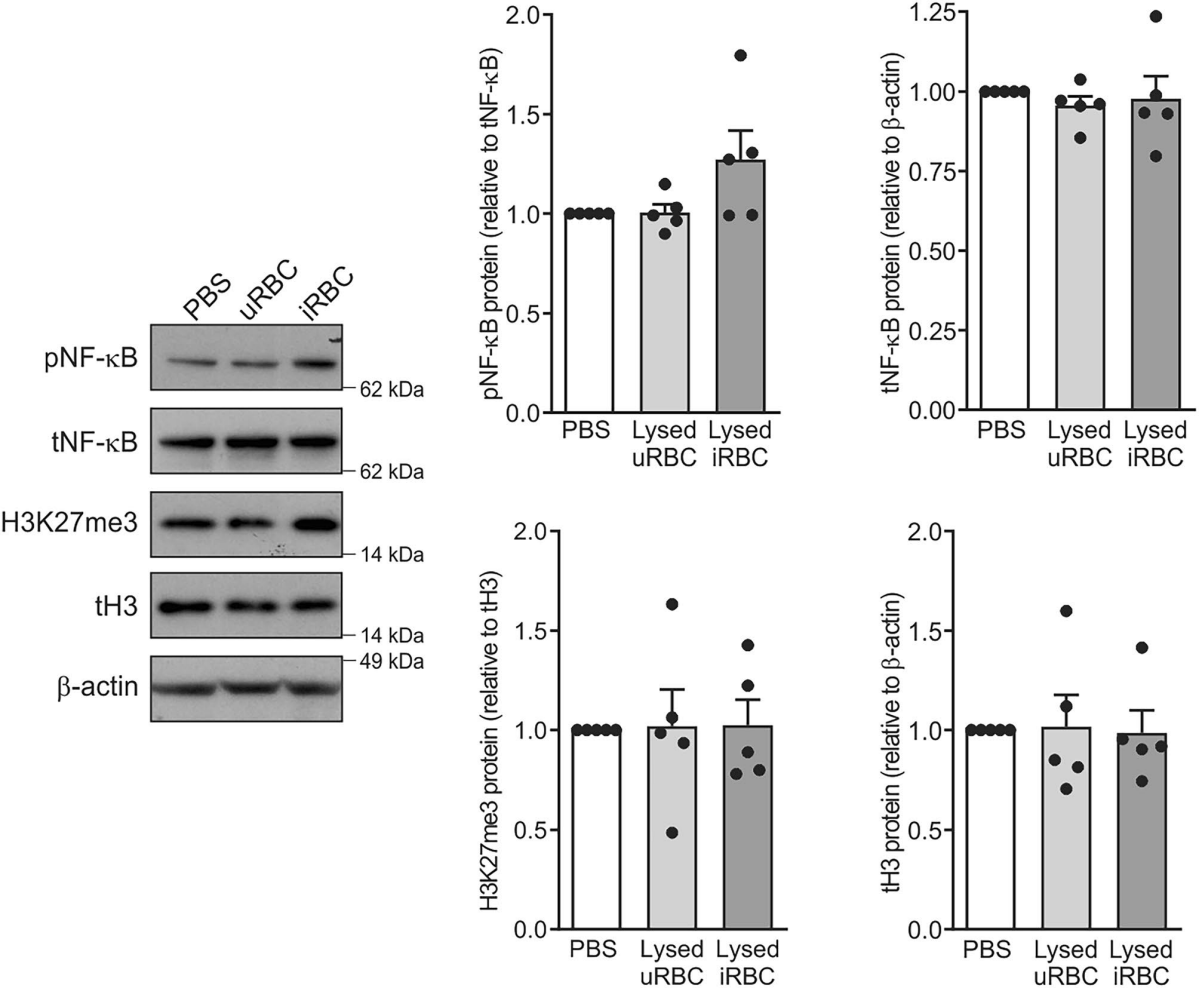
Lysed *P. falciparum*-infected RBC marginally regulate NF-κB phosphorylation but not H3K27 trimethylation. Monocytes (1.5 × 10^6^) were incubated with lysed uninfected red blood cells (uRBC) or lysed *P. falciparum*-infected red blood cells (iRBC) for 4 h, with untreated monocytes (PBS) serving as a negative control. Whole cell lysates were run on a 10% NuPAGE Bis–Tris protein gel and then subjected to Western blotting with antibodies against phosphorylated p65 NF-κB (pNF-κB), total p65 NF-κB (tNF-κB), trimethylated histone 3 lysine 27 (H3K27me3), total histone 3 (tH3) and β-actin proteins. A representative Western blot is shown together with quantified proteins using densitometry and normalized relative to their expression in PBS control, which was given an arbitrary value of 1. Results are graphed as scatter plots with bars indicating mean ± SEM. P-values were calculated using one-way ANOVA with Dunnett’s multiple comparison test to compare each condition to the untreated control (n = 5 donors). Original Western blots are presented in Supplementary Fig. 1.

We next investigated the molecular pathways that may be activated by lysed iRBC in monocytes. Inflammatory cytokines, including TNF and IL-6, are known to be controlled by NF-kB transcription factor, and the expression of these genes is basally suppressed by the repressive histone 3 lysine 27 trimethylation (H3K27me3) mark^17,18^. Monocytes were treated with PBS, lysed uninfected red blood cells (uRBC) or lysed iRBC for 4 h. While there was a marginal increase in the levels of phosphorylated p65 NF-kB (pNF-kB), there were no changes in the H3K27me3 levels in monocytes following exposure to lysed iRBC compared to monocytes treated with either PBS control or uRBC, as assessed by Western blotting (Fig. 2). These findings suggested that, in addition to NF-kB transcriptional activation, epigenetic modifications other than H3K27me3 may be involved in regulating cytokine production in iRBC-exposed monocytes.

### Increased levels of H3K4 methylation are found in *P. falciparum*-exposed monocytes

**Figure 3:**
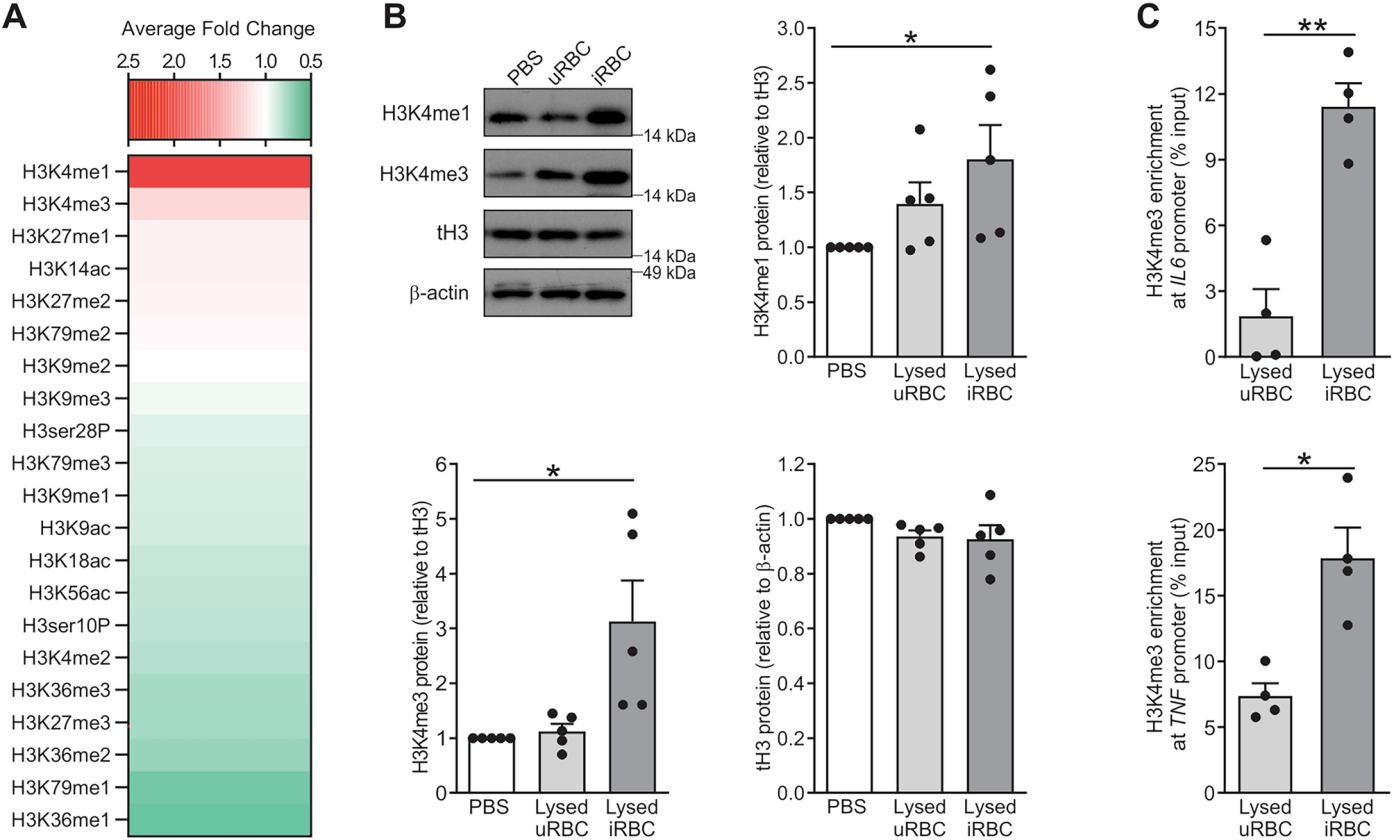
Increased H3K4 methylation are found in *P. falciparum*-exposed monocytes. (**A**) Monocytes (2.5 × 10^6^) were exposed to either lysed uninfected red blood cells (uRBC) or lysed infected red blood cells (iRBC) for 4 h before histone enrichment and analyses. Histone modifications from two donors were normalized respective to their total histone 3 expression and were expressed as fold change of iRBC versus uRBC. A fold-change greater than 1.0 indicates an increase in histone modification (red) in iRBC-treated monocytes versus that in uRBC-treated monocytes. (**B**) Monocytes (1.5 × 10^6^) were exposed to either lysed uRBC or lysed iRBC for 4 h, with untreated monocytes (PBS) serving as a negative control. Whole cell lysates were run on a 12% NuPAGE Bis–Tris protein gel and then subjected to Western blotting with antibodies against monomethylated histone 3 lysine 4 (H3K4me1), trimethylated histone 3 lysine 4 (H3K4me3), total histone 3 (tH3), and β-actin proteins. A representative Western blot is shown together with quantified proteins using densitometry and normalized relative to their expression in untreated monocyte control, which was given an arbitrary value of 1. (**C**) Monocytes were treated with either uRBC or iRBC for 4 h. ChIP analysis of the association of H3K4me3 with the promotor regions of the *TNF* and *IL-6* genes is expressed as a percentage of the input DNA (n = 4). Results are graphed as scatter plots with bars indicating mean ± SEM. P-values were obtained using either one-way ANOVA with Dunnett’s multiple comparison test to compare each condition to the PBS control (**A**,**B**) or paired t-test (**C**), where *P < 0.05, **P < 0.01 (n = 4–5 donors). Original Western blots are presented in Supplementary Fig. 2.

To determine broadly if histones are modified in monocytes upon exposure to *P. falciparum*, a histone 3 modification multiplex assay was performed. Out of the 21 known histone 3 modifications that were assayed, the monomethylated histone 3 lysine 4 (H3K4me1) and trimethylated histone 3 lysine 4 (H3K4me3) marks were the two most highly upregulated modifications in lysed iRBC-exposed monocytes versus lysed uRBC-exposed monocytes, with an average fold-change of 2.07 and 1.2, respectively (Fig. 3A). These two histone modifications were further validated by Western blotting, where significant increase in the levels of H3K4me1 and H3K4me3 were observed in lysed iRBC-exposed monocytes and as expected, the total H3 levels remained unchanged across all conditions (Fig. 3B). Methylation of H3K4, in particular H3K4me3, has previously been associated with active transcription of the TNF and IL-6 genes^16^, and therefore, we performed a chromatin immunoprecipitation assay. Monocytes exposed to lysed iRBC contained enriched H3K4me3 in the promoter regions of TNF and IL-6 genes compared to those exposed to control uRBC (Fig. 3C).

### Pharmacological inhibitor of H3K4 methylation, WDR5-0103, suppresses TNF and IL-6 production by *P. falciparum*-exposed monocytes

**Figure 4:**
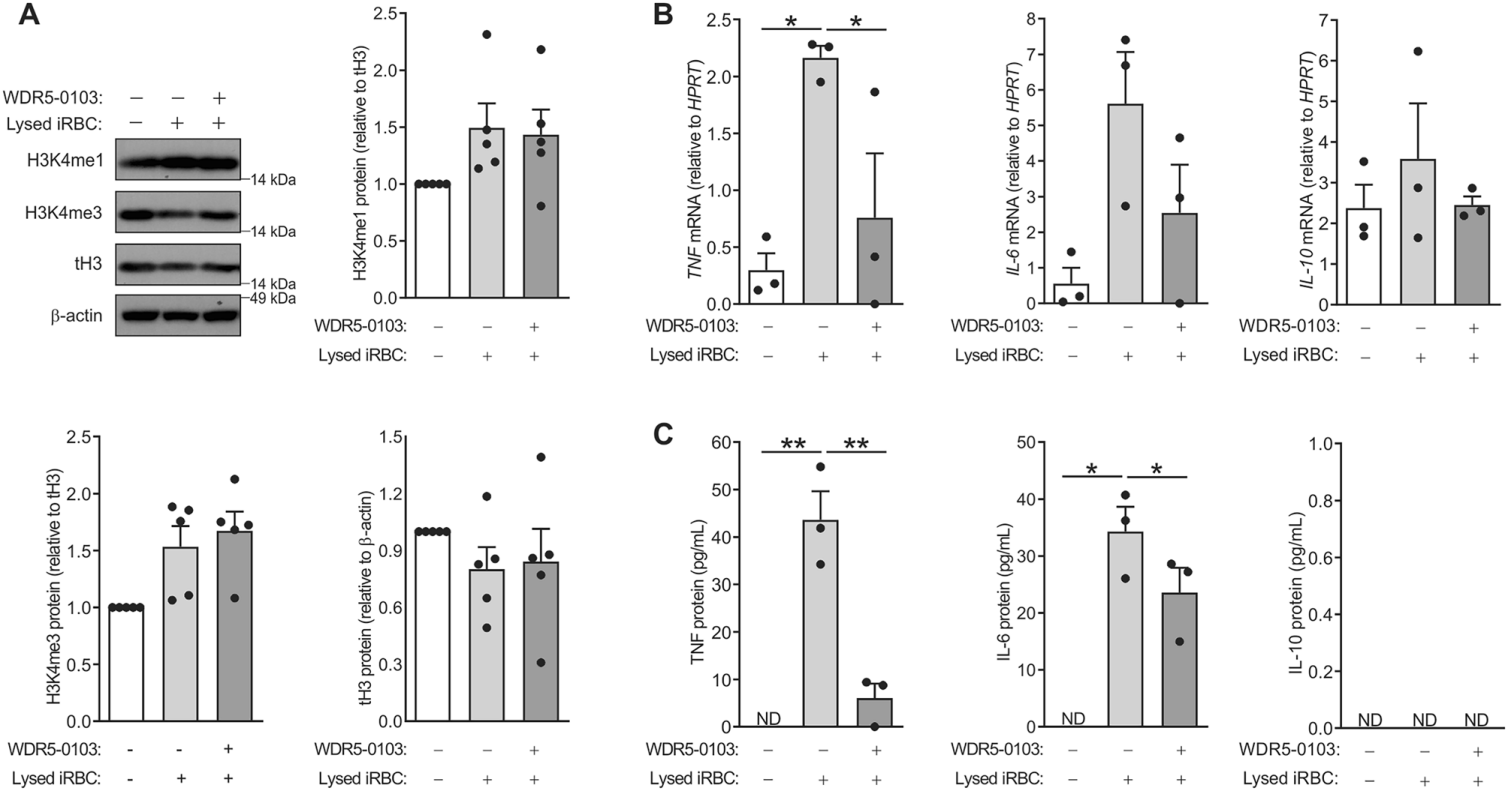
Pharmacological inhibitor of H3K4 methylation, WDR5-0103, suppresses TNF and IL-6 production by *P. falciparum*-exposed monocytes. Monocytes were pretreated with the H3K4 methylation inhibitor WDR5-0103 (100 µM) for 16 h then incubated with lysed infected red blood cells (iRBC) for 4 h, with untreated monocytes serving as a negative control. (**A**) Whole cell lysates were run on a 10% NuPAGE Bis–Tris protein gel and then subjected to Western blotting with antibodies against monomethylated histone 3 lysine 4 (H3K4me1), trimethylated histone 3 lysine 4 (H3K4me3), total histone 3 (tH3) and β-actin proteins. A representative Western blot is shown together with quantified proteins, using densitometry and normalized relative to their expression in control, which was given an arbitrary value of 1 (n = 5 donors). (**B**) *TNF, IL-6* and *IL-10* mRNA expression by qPCR (n = 3 donors). (**C**) Secreted TNF, IL-6 and IL-10 proteins by ELISA (n = 3 donors). Results are graphed as scatter plots with bars indicating mean ± SEM. P-values were obtained using one-way ANOVA with Tukey’s multiple comparison test to compare each condition to the PBS control, where *P < 0.05, **P < 0.01. *ND* not detected. Original Western blots are presented in Supplementary Fig. 3.

The SET1 family of histone methyltransferases (HMTs) are known for their role in the methylation of H3K4 and WDR5-103, an inhibitor specific for SET1 HMTs, has been shown to have no inhibitory effects on other HMTs, including SETD7, G9a, EHMT1, SUV39H2, SETD8, PRMT3 and PRMT5, at concentrations up to 100 µM^20^. To examine whether H3K4 methylation in *P. falciparum*-exposed monocytes could regulate cytokine production, we pre-treated monocytes with 100 μM of WDR5-0103 or vehicle DMSO for 16 h, before cells were exposed to lysed iRBCs for another 4 h. Although there was an increase in the levels of mono- and tri-methylated H3K4 in *P. falciparum*-exposed monocytes, this global upregulation was not suppressed when monocytes were pre-treated with WDR5-0103 (Fig. 4A). Next, we examined whether the H3K4 methyltransferase inhibitor had any impact on the *P. falciparum*-induced cytokine production by monocytes. Pre-treatment of monocytes with WDR5-0103 resulted in a marked decrease in TNF and IL-6 mRNA (Fig. 4B) and protein (Fig. 4C) levels. On the other hand, the inhibitor did not downregulate IL-10 mRNA (Fig. 4B), and consistent with the data on the IL-10 protein in Fig. 1B, the secreted protein could not be detected in monocyte culture supernatants following 4 h of exposure to iRBC (Fig. 4C). Taken together, treating monocytes with WDR5-0103 did not appear to affect global methylation status of H3K4, but consistently suppressed TNF and IL-6 expression, suggesting a role for H3K4 methylation in *P. falciparum*-induced proinflammatory cytokine production by monocytes.

## Discussion

While *P. falciparum* can trigger cytokine production by immune cells as part of host defense, uncontrolled excessive cytokine production can lead to severe malaria symptoms, further exacerbating the disease^3^. To study the epigenetic and transcriptional regulators of monocyte cytokine production during blood-stage infection, we established an in vitro system using monocytes from healthy donors. With this model we found at both the mRNA and protein levels that monocytes exposed to lysed iRBC showed robust inflammatory cytokine responses relative to monocytes exposed to either live iRBC or Hz.

The more robust response to lysed iRBC compared to live iRBC may be because live parasites have the *P. falciparum* erythrocyte membrane protein 1 (P*f*EMP1) antigen expressed on the surface of the RBC. Indeed, it has been previously shown that human monocytes treated with P*f*EMP1-containing parasites released less TNF, IL-1β, IL-6 and IL-10 compared to monocytes exposed to parasites where the P*f*EMP1 was not expressed on the surface^5^. Significantly, individuals from malaria-endemic regions produce P*f*EMP1-specific antibodies that can engage Fc receptors and also express the CD36 scavenger receptor on the monocyte surface, which can directly bind certain variants of P*f*EMP1^21,22^. Several approaches can be undertaken to replicate iRBC detection and cytokine response of patients from malaria-endemic regions: firstly, opsonizing the live iRBC with malaria specific antibodies or complement factors to increase the range of detection with additional Fcγ and complement receptors^23^, secondly, using monocytes from individuals previously exposed to malaria who may have Toll-like receptors (TLRs) with polymorphisms which alter the detection of PAMPs associated with the malaria parasite^24^ and lastly, using an alternative parasite strain as the CS2 strain used in this study binds to chondroitin sulfate A in the placenta of pregnant women and not to the CD36 receptors^25,26^.

Hemozoin is a hydrophobic, insoluble crystal formed during the digestion of hemoglobin by *Plasmodium* parasites^27^. In our in vitro system, the purified Hz failed to elicit a robust cytokine response in monocytes. While previous studies have suggested a role for Hz in promoting pro-inflammatory cytokine response on blood mononuclear cells, including monocytes and macrophages, it has been recognized that Hz is inert, rather Hz-coupled parasite DNA is responsible for cell activation^22,28^. Because the Hz used in our study was not synthetic, instead prepared from live iRBC, it is possible that other parasite components that could be present might have contributed to a weak cytokine response.

During the intraerythrocytic stage, the parasites increase in size before morphing into trophozoites, which further divide into schizonts that eventually lyse releasing merozoites to invade more erythrocytes. Schizont rupture releases toxins that can trigger the symptoms of malaria infection^29^. In our in vitro system, the lysed iRBC mimics the process of schizont rupture. The potent cytokine response by monocytes exposed to lysed iRBC could be triggered by a cocktail of released internal parasite antigens. Furthermore, released Hz from lysed iRBC could be contaminated with other pathogen-associated molecular patterns (PAMPs), such as glycosylphosphatidylinositols and DNA, and therefore could have triggered a robust cytokine response compared to our in vitro purified Hz. These malaria parasite PAMPs can be sensed by TLRs, activating many signaling cascades, including the NF-kB pathway that may be responsible for regulating a robust pro-inflammatory cytokine response by monocytes^22,30^. In contrast to early TNF and IL-6 production, secreted IL-10 was only detected in monocyte culture supernatants 24 h after exposure to iRBCs. Since IL-10 acts as an anti-inflammatory cytokine, restricting the overproduction of pro-inflammatory cytokines, the observed delayed formation of IL-10 is consistent with its function^31^, further validating our in vitro system.

The NF-κB transcription factor family consists of five proteins, namely p65 (RelA), RelB, c-Rel, p105/p50 (NF-κB1), p100/52 (NF-κB2), that bind to each other to form transcriptionally active homo- or heterodimeric complexes^32^. This family of transcription factors plays a key role in regulating gene expression of many pro-inflammatory cytokines, including TNF and IL-6. In unstimulated cells, NF-κB is bound to inhibitor kappa B (IκB) protein and remains in the cytoplasm in an inactive form. Upon stimulation of cells (e.g., with LPS), IκB is phosphorylated by IκB kinase and then rapidly degraded by the proteosome. Subsequently, freed NF-κB dimer complexes translocate into the nucleus, where they promote gene transcription. While our studies found only a marginal increase in the phosphorylation of p65 NF-κB subunit in monocytes, several other studies have shown *P. falciparum* can activate NF-κB and its subsequent translocation to the nucleus in macrophages and blood mononuclear cells^33^.

Gene transcription is finely controlled by a dynamic interplay between activating and repressing modifications of histone tails^34^. It is known that lysine and arginine methylations can be facilitated by histone methyl transferases (HMT), while their demethylation can be catalyzed by demethylases^35^. For example, presence of a repressive H3K27me3 in the promoter region of a gene leads to transcriptional inactivation via formation of heterochromatic regions. Significantly, an NF-κB responsive histone demethylase JMJD3 has been shown to remove the repressive H3K27me3 mark that basally suppresses NF-κB inducible genes in LPS-treated macrophages^36^. In our study, despite an increase in the transcripts of TNF, IL-6 and IL-10 genes, no change in the global trimethylated H3K27 levels were observed between monocytes exposed to lysed uRBC and iRBC at 4 h, suggesting that removal of H3K27me3 could perhaps be specific for the respective cytokine gene promoters. Furthermore, only 16% of LPS-responsive genes displayed a decrease in H3K27me3 following 4-h LPS treatment of macrophages, indicating that other activating histone modifications may contribute to the regulation of the remaining genes^36^. Indeed, macrophages have been shown to gain activating H3K4 methylation and H3K9 acetylation epigenetic marks to increase TNF and IL-1β production in response to *Leishmania donovani* and *Toxoplasma gondii* infections^37,38^*.* We identified an increase in H3K4 mono- and trimethylation in monocytes exposed to lysed iRBC. These histone modifications are known as positive gene regulators, with H3K4me1 working on distal enhancers and H3K4me3 found at transcription start sites^39,40^. We also demonstrated that there was an increase in the active H3K4me3 mark in the promoter regions of the *TNF* and *IL-6* genes in monocytes exposed to iRBC compared to those exposed to control uRBC. Consistent with our finding, a recent study by *Guha *et al., found that naïve monocytes exposed in vitro to live opsonized iRBCs exhibited elevated levels H3K4me3 on the promoters of TNF and IL-6 genes, corresponding to their increased secretion^16^. Significantly, monocytes isolated from individuals infected with *P. falciparum* also showed increased H3K4me3 and cytokine production^41,42^, highlighting the clinical significance of H3K4me3 modifications and implicating them in pro-inflammatory cytokine production and associated pathology.

Methylation of histone lysine residues by methyltransferases is implicated in several human diseases, especially cancer, and therefore HMT have been identified as potential therapeutic targets. The SET1 family of HMT complex contain four integral components, namely WD (tryptophan (W)–aspartic acid (D) dipeptide) repeat containing protein 5 (WDR5), Retinoblastoma Binding Protein 5 (RbBP5), Absent, Small or Homeotic 2-like protein (ASH2L) and Dumpy-30 (Dpy30) are known for their involvement in H3K4 methylation^43^. The enzymatic activity of the SET1 family can be inhibited by targeting WDR5 (for example, with WDR5-0103 drug), which disrupts the formation of the HMT complex and subsequently prevents H3K4 methylation^44^. In the current study, treatment of lysed iRBC-exposed monocytes with WDR5-0103 did not affect the global H3K4 methylation levels, but significantly inhibited the production of pro-inflammatory cytokines, TNF and IL-6. Our findings are in parallel with other studies^45–47^, where WDR5 antagonists have been shown not to change global H3K4 methylation but lead to downregulation of TNF and IL-6 by perhaps specifically inhibiting the histone modification in their respective gene promoters. Furthermore, WDR5-0103 had no effect on IL-10 transcripts, and the IL-10 protein could not be detected in the culture supernatants from monocytes exposed to iRBC for 4 h, suggesting a direct role of H3K4 methylation in regulating the proinflammatory cytokines, as opposed to the anti-inflammatory IL-10 modulating their levels in an autocrine loop.

In summary, monocytes are essential for host protection against malaria through cytokine production and phagocytosis; however, excessive production of inflammatory cytokines can result in systematic inflammation. Our findings offer mechanistic insights into how monocytes mount an inflammatory cytokine response upon exposure to *P. falciparum*. Targeting H3K4 methylation with WDR5 antagonists can be useful in controlling excessive inflammatory TNF and IL-6 production and thereby managing the pathogenesis of severe malaria.

## Materials and methods

### Isolation of human monocytes from buffy coat

Buffy coats were sourced from the Australian Red Cross Lifeblood as approved by the University of Melbourne Human Research Ethics Committee (2021-20542) and all methods were performed in accordance with the relevant guidelines and regulations. Informed consent was obtained from all subjects and/or their legal guardian(s). Human monocytes were isolated by negative selection using the RosetteSep Human Monocyte Enrichment cocktail (Stem Cell Technologies, Vancouver, BC, Canada), as before^48^. Negatively selected monocytes were further enriched by performing two rounds of ‘platelet wash’ with centrifugation at 120×*g* for 10 min at room temperature with no brake. The purity of the enriched monocytes was found to be consistently above 85%, as ascertained by flow cytometry with anti-human CD14-PerCP-Cy5.5 antibodies and IgG1k-PecCP-Cy5.5 as isotype control (Thermo Fischer Scientific, Waltham, MA). Isolated monocytes were resuspended in 10 mL of complete RPMI 1640 culture medium, containing 10% heat inactivated fetal calf serum, 100 U/mL penicillin, 100 mg/mL streptomycin, and 1% GlutaMax-1 (Life Technologies, Carlsbad, CA). Cell count and viability were assessed using Trypan blue staining and counted via Countess II Automated Cell Counter (Thermo Fischer Scientific). Monocytes were cultured with *P. falciparum*-iRBC or malaria pigment Hz at 37 °C in a 5% CO_2_ humidified environment.

### Preparation of live and lysed *P. falciparum*-infected red blood cells

*P. falciparum* parasites (CS2 strain) were cultured in vitro in O-positive red blood cells in RPMI 1640-4-(2-hydroxyethyl)-1-piperazineethanesulfonic acid (Life Technologies), supplemented with 0.2% w/v sodium bicarbonate, 5% heat-inactivated human serum (Australian Red Cross Lifeblood), and 0.5% Albumax II (Life Technologies), as before^49^. Parasite cultures were tested for mycoplasma contamination using the MycoAlert kit (Lonza, Basel, Switzerland). Trophozoite-stage iRBC were enriched to 85–95% purity using Percoll density gradient (Amersham Biosciences, Amersham, UK). Live trophozoite-enriched cultures were collected and used as treatment for monocyte cultures or stored at − 80 °C. Frozen trophozoite stage iRBC underwent two freeze–thaw cycles to prepare lysed parasites and then were stored at − 30 °C until used for treatment or Hz extraction. Control uninfected red blood cells (uRBC) were sourced from Australian Red Cross Lifeblood and further purified by washing thrice with PBS (pH 7.4). The uRBC were resuspended in RPMI 1640 media and stored at − 30 °C until used.

### Hemozoin isolation

Lysed iRBC were incubated with a final concentration of 0.1% saponin for 2 min at RT. The samples were then sonicated twice using the Bioruptor® XL (Diagenode, Denville, NJ) for 30 s on high speed, then spun down at 11,000 × g for 30 min at RT. Following this, the pellet was washed thrice with 100 mM NaHCO_3_, 2% SDS (pH 9), followed by centrifugation (11,000×*g*, 2 min, RT). The washed pellet was sonicated again for 30 s at high speed. The lysate was centrifuged at 11,000×*g* for 30 min at RT then washed twice with 2% SDS (pH 9), then twice again with purified or Milli-Q water. The isolated Hz was resuspended in distilled water and stored at − 80 °C.

### Quantitative PCR

Extraction of total RNA was performed using Isolate II RNA mini kit (Bioline, London, UK). The RNA concentration and purity were measured using NanoDrop One C spectrophotometer (Nanodrop Technologies, Anaheim, USA). The resulting RNA concentrations were standardized using the lowest sample concentration and cDNA was made using the Tetro Reverse Transcriptase (Bioline). Quantitative PCR (qPCR) was performed using the QuantStudio 5 Real-Time PCR System (Thermo Fischer Scientific) with predeveloped TaqMan probe/primer combinations for human TNF (HS00174128), IL-6 (Hs00174131), IL-10 (Hs00961622) and internal control hypoxanthine phosphoribosyl transferase 1 (HPRT1) (4325801, Thermo Fischer Scientific). Threshold cycle numbers were transformed to cycle threshold values, and the results were plotted using GraphPad Prism version 9.5.

### ELISA

Secreted TNF, IL-6 and IL-10 in culture supernatants were measured by respective ELISA kits as per manufacturer’s instructions (MabTech, Nacka Strand, Sweden).

### Western blotting

Western blotting was performed as described previously^48^. Briefly, whole cell extracts from monocytes were lysed with RIPA buffer. The protein concentrations were measured with a Bio-Rad Bradford protein assay kit using the SmartSpec 3000 spectrometer (Bio-Rad, Hercules, CA). Equal amounts of whole cell lysates were run on a 10 or 12% NuPAGE Bis–Tris Protein Gel (Life Technologies). The separated proteins were transferred onto a polyvinylidene fluoride (PVDF) membrane (Merck Millipore, Burlington, MA). Following transfer, membranes were stained with Ponceau S (Sigma-Aldrich, St. Louis, MO) to visualize protein bands and cut into three sections based on molecular weight ladder (6–28 kDa, 28–49 kDa and 49–98 kDa) to simultaneously probe with antibodies detecting various sizes of interested proteins. Antibodies were against human total p65 NF-κB, phosphorylated p65 NF-κB (Cell Signaling Technologies, Danvers, MA), histone 3 lysine 27 trimethylation (H3K27me3), histone 3 lysine 4 monomethylation (H3K4me1), histone 3 lysine 4 trimethylation (H3K4me3), total histone 3 (tH3) (Merck Millipore) and β-actin (Sigma-Aldrich). Western blots were quantified by densitometry using Bio-Rad GS-800 calibrated imaging densitometer with Quantity One software version 4.6.9.

### Histone 3 modification multiplex assay

Total histones from monocyte cultures were extracted using the Histone Extraction Kit (ab113476, Abcam, Cambridge, UK) as per manufacturer’s instructions. The extracted histones were subjected to Colorimetric Histone H3 Modification Multiplex Assay using a kit (ab185910, Abcam), containing 21 known histone modifications, following manufacturer’s instructions. Histone modification was calculated by subtracting the reference wavelength (OD measured at 655 nm) from the test wavelength (OD measured at 450 nm). The resulting sample ODs were standardized to their respective tH3 reading before being expressed as fold change of iRBC-treated monocytes over uRBC-treated monocytes.

### ChIP-qPCR assay

Chromatin immunoprecipitation (ChIP) assays were performed as described previously^50^. Briefly, monocytes (5 × 10^6^) were treated with either uRBC or iRBC for 4 h before crosslinking protein-DNA complexes with 1% formaldehyde for 10 min at room temperature. ChIP was performed using a SimpleChIP Plus Sonication Chromatin IP Kit (Cell Signaling Technologies) according to the manufacturer’s instructions. DNA was sheared with Bioruptor XL (Diagenode) with a HI pulse setting and 30 s on and 30 s off pulses. The cycle was repeated 30 times, resulting in a sonication time of 30 min in total to achieve chromatin fragments of 200 to 1,200 base pairs. Chromatin immunoprecipitation was performed with 1 μg of anti-H3K4me3 or anti-histone H3 antibodies (Millipore), followed by reversal of cross-linking. Immunoprecipitated DNA was purified with the MinElute PCR purification kit (QIAGEN). The quantitative PCR reaction was then performed on the immunoprecipitated DNA fragments with a SensiFAST SYBR Hi-ROX Kit (Bioline) and the following primer pairs: *IL-6*, forward 5′-AGCTCTATCTCCCCTCCAGG-3′ and reverse 5′-ACACCCCTCCCTCACACAG-3′; *TNF*, forward 5′- CAGGCAGGTTCTCTTCCTCT-3′ and reverse 5′- GCTTTCAGTGCTCATGGTGT-3′^16^. Enrichment of H3Kme3 in the promoter regions of the *IL-6* and *TNF* genes was expressed as a percentage of the input DNA.

### Statistics

Statistical analyses between two groups were performed using paired t-test and multiple groups with a one-way ANOVA with Dunnett’s or Tukey’s multiple comparisons test. The *p* values < 0.05 were considered significant. Data were plotted as mean ± S.E.M. from at least three independent experiments using GraphPad Prism version 9.5.

### Supplementary Information

Supplementary Figures.

## Data Availability

All generated or analyzed data during this study are included in this paper.
