# Supplementary material for: Epigenetic and transcriptional regulation of cytokine production by Plasmodium falciparum-exposed monocytes
Source: Sci Rep. 2024 Feb 5;14:2949. doi: 10.1038/s41598-024-53519-w (PMC10844200; doi:10.1038/s41598-024-53519-w)
Supplement: Supplementary file 1 — Supplementary Figures. [file 41598_2024_53519_MOESM1_ESM.pdf]

Supplementary Figure 1

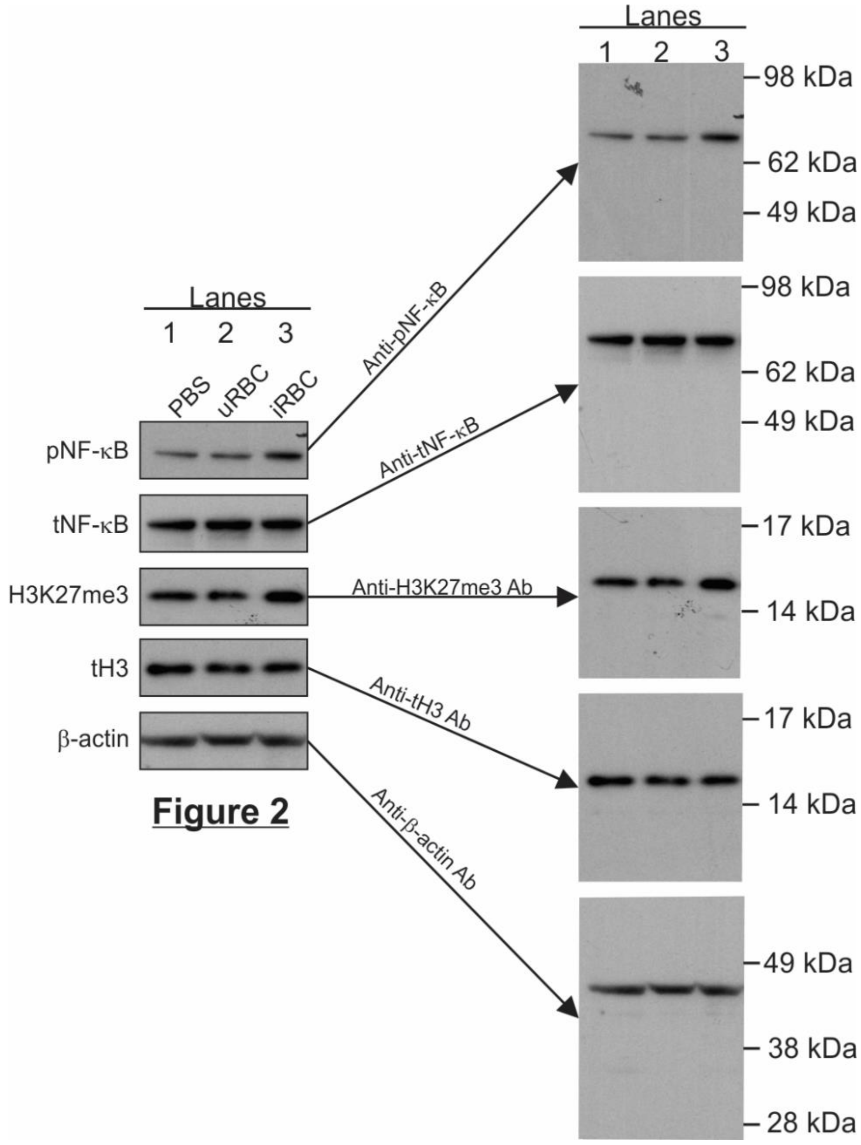

Supplementary Figure 1: Lysed *P. falciparum*-infected RBC marginally regulate NF-κB phosphorylation but not H3K27 trimethylation.

Monocytes (1.5 × 10^6) were incubated with lysed uninfected red blood cells (uRBC) or lysed *P. falciparum*-infected red blood cells (iRBC) for 4 hours, with untreated monocytes (PBS) serving as a negative control. Whole cell lysates were subjected to Western blotting with antibodies against phosphorylated p65 NF-κB (pNF-κB), total p65 NF-κB (tNF-κB), trimethylated histone 3 lysine 27 (H3K27me3), total histone 3 (tH3) and β-actin proteins. Both cropped (**Figure 2**) and original Western blots (membranes were cropped to into sections, based on molecular weight ladder, prior to hybridization to simultaneously probe with antibodies detecting various sizes of interested proteins) are shown.

Supplementary Figure 2

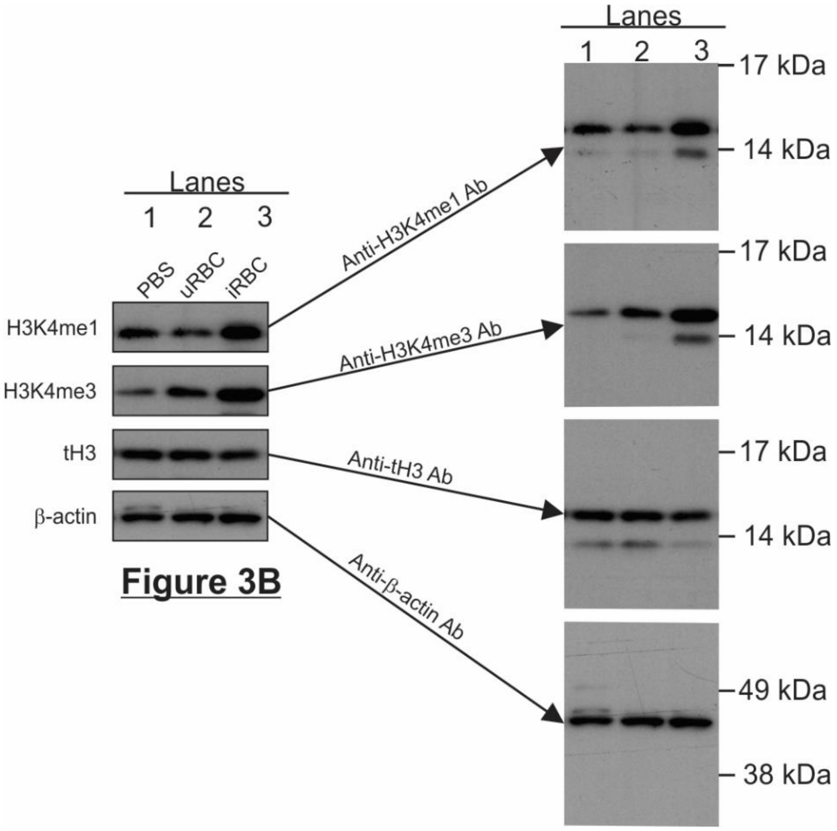

Supplementary Figure 2: Increased levels of mono- and tri-methylated H3K4 are found in *P. falciparum*-exposed monocytes.

Monocytes (1.5×10^6) were exposed to either lysed uRBC or lysed iRBC for 4 h, with untreated monocytes (PBS) serving as a negative control. Whole cell lysates were subjected to Western blotting with antibodies against monomethylated histone 3 lysine 4 (H3K4me1), trimethylated histone 3 lysine 4 (H3K4me3), total histone 3 (tH3), and β-actin proteins. Both cropped (**Figure 3B**) and original Western blots (membranes were cropped to into sections, based on molecular weight ladder, prior to hybridization to simultaneously probe with antibodies detecting various sizes of interested proteins) are shown.

Supplementary Figure 3

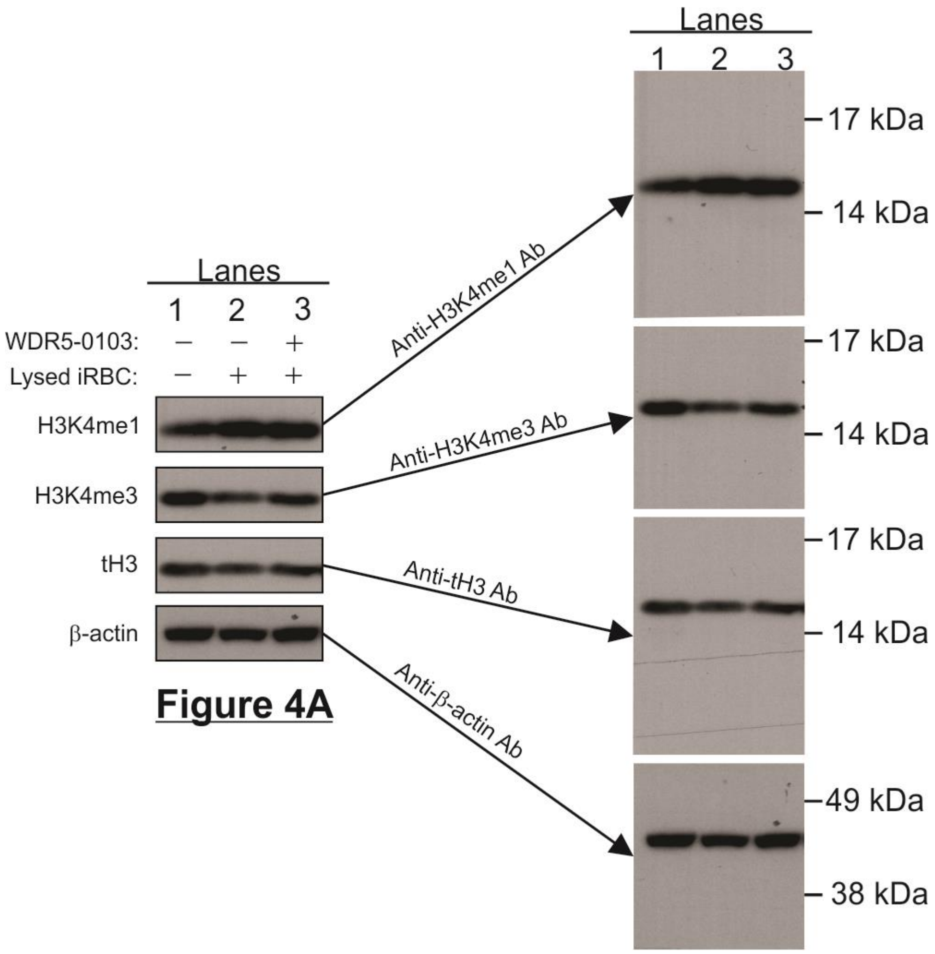

Supplementary Figure 3: Pharmacological inhibitor of H3K4 methylation, WDR5-0103, suppresses TNF and IL-6 production by *P. falciparum*-exposed monocytes.

Monocytes were pretreated with the H3K4 methylation inhibitor WDR5-0103 (100 μM) for 16 h then incubated with lysed infected red blood cells (iRBC) for 4 h, with untreated monocytes serving as a negative control. Whole cell lysates were subjected to Western blotting with antibodies against monomethylated histone 3 lysine 4 (H3K4me1), trimethylated histone 3 lysine 4 (H3K4me3), total histone 3 (tH3) and β-actin proteins. Both cropped (**Figure 4A**) and original Western blots (membranes were cropped to into sections, based on molecular weight ladder, prior to hybridization to simultaneously probe with antibodies detecting various sizes of interested proteins) are shown.
